# A 90 GHz Broadband Balanced 8-Way Power Amplifier for High Precision FMCW Radar Sensors in 65-nm CMOS

**DOI:** 10.3390/s22093114

**Published:** 2022-04-19

**Authors:** Hyeonseok Lee, Van-Son Trinh, Jung-Dong Park

**Affiliations:** Division of Electronics and Electrical Engineering, Dongguk University, Seoul 04620, Korea; leehyeonseok@dgu.ac.kr (H.L.); trinhvanson92@dongguk.edu (V.-S.T.)

**Keywords:** frequency-modulated continuous-wave (FMCW), CMOS, broadband, transformer-based combiner, power amplifier (PA), W-band, hybrid coupler

## Abstract

We present a W-band 8-way wideband power amplifier (PA) for a high precision frequency modulated continuous wave (FMCW) radar in 65-nm CMOS technology. To achieve a broadband operation with an improved output power for a high range resolution and high distance coverage of FMCW radar sensors, a balanced architecture is employed with the Lange coupler which naturally combines the output powers from two 4-way push-pull PAs. By utilizing a transformer-based push-pull structure with a cross-coupled capacitive neutralization technique, the gate-drain capacitance of the 4-way PA is compensated for the stabilization with an improved power gain. Interstage matching was performed with transformers for a reduced loss from the matching network and minimal area occupation. The implemented balanced 8-way PA achieved a saturated output power (*P_sat_*) of 16.5 dBm, a 1-dB compressed output power (OP_1dB_) of 13.3 dBm, a power-added efficiency (PAE) of 9.9% at 90 GHz and 3-dB power bandwidth was 20.4 GHz (79.2–99.6 GHz).

## 1. Introduction

Recently, frequency-modulated continuous-wave (FMCW) radar sensors have been widely used for airborne radars, automotive cruise control, and weather radars. FMCW radar sensors that operate at millimeter-wave can achieve better spatial and range resolution owing to their wide sweep bandwidth, as well as a sharper beamwidth for a given antenna size. Moreover, low oxygen attenuation and availability of small size antennas at W-band are appealing in improving propagation loss and implementing a coherent multi-receiver integration for better sensitivity [1]. In this regard, one of the most crucial parameters of a high-precision FMCW radar is a sweep bandwidth that improves high range resolution, e.g., FMCW radar sweep bandwidth should be as wide as 10 GHz to distinguish objects that are 1.5 cm apart [2], which is around 10% of the fractional bandwidth of a 90 GHz FMCW radar sensor.

With the advancement of CMOS technology, a bulk silicon-based CMOS process has been considered one of the most popular technologies for mass production, owing to its low-cost and high-level integration features. However, the design of the wideband power amplifier in a nanoscale CMOS is quite challenging due to the serious channel length modulation effect in the nanoscale CMOS, which directly determines the output power, linearity, and power gain. Moreover, the reduction of the maximum available gain (MAG) and maximum stable gain (MSG) with the increase in operating frequency correspondingly degrades the power gain and efficiency and it hinders the gain flatness to achieve wide bandwidth for FMCW radar applications at the millimeter-wave regime.

Parasitic capacitances in RF transistors have been identified as the main factor of the MAG and MSG reduction. To compensate for parasitic capacitances, the common source (CS) topology with the capacitive cross-coupling neutralization method has been widely applied at millimeter-wave amplifiers [3]. The gate-drain capacitance *C_gd_* constructs undesirable shunt feedback which decreases the gain and the stability of systems. This can be relieved by using cross-coupled neutralization capacitors *C_n_* between the drain node of one side of the device and the gate node of the other side in a push-pull structure. Additionally, a value of *C_n_* should be carefully chosen, since *C_n_* can create positive feedback when the capacitive neutralization is over-compensated. Thus, the power gain and power-added efficiency (PAE) should be carefully compromised with stability.

In designing millimeter-wave CMOS PAs, one of the most important parameters is the saturated output power (*P_sat_*) which is determined by an available active device size considering a feasible output matching network. Since CMOS PAs are usually implemented with a low supply voltage of around 1 V due to low breakdown voltages from the thin oxide layer in MOS, it is hard to achieve an efficient output power matching network with a relatively large size device. To resolve this issue, there have been enormous efforts in the literature. By stacking the MOS transistor, the output voltage can be scaled up but necessitates an expensive SOI technology if the required stack is more than two [4]. Various power combining techniques have been more prevalently used to boost the output power of a PA. Transmission-line-based combiner [5,6], λ/4-based combiner [7,8], and transformer-based combiner [9,10,11,12,13] are widely used to boost the output power. Among these approaches, the transformer-based power combiner is considered the widely adopted technique due to its simplified matching network, and no additional AC coupling capacitor is required, which has a low-quality factor at the millimeter-wave regime. It should be noticed that the 3-dB power bandwidth of the employed PA must satisfy the sweep bandwidth requirement. Moreover, the flatness variation in the output power level should be kept minimized to avoid sidelobes from the unwanted amplitude modulation.

In this paper, a wideband balanced 8-way power amplifier with the Lange couplers is presented by combining two 4-way transformer-based push-pull PAs for advanced performances in the output power, efficiency, and wide power bandwidth with improved flatness. The W-band PA was implemented in TSMC 65 nm CMOS, and it achieved the output power of 16.4 dBm with 26.7 dB gain, 9.8% PAE at 91.2 GHz, 13 GHz (83–96 GHz) of 3-dB gain bandwidth, more than 20 GHz of 3-dB output power bandwidth, and 1.5 dB of variation in the saturated output power. Analysis of choosing a proper neutralization capacitor was performed considering the stability factor, MAG, and MSG of the transistor. Moreover, the transformer-based matching networks and hybrid combiner were investigated to achieve a high output power and PAE with improved 3-dB power bandwidth at W-band. By utilizing a hybrid combining technique together with transformer-based push-pull PAs, the proposed PA achieves improved output power, gain, and bandwidth, which results in the highest Figure-of-Merit (FoM) among the recently reported CMOS PAs operating at the 90 GHz region. Moreover, the presented PA is robust to the load variations owing to the balanced configuration.

## 2. Design of 4-Way Push-Pull Power Amplifier

The proposed balanced 8-way PA consists of the two transformer-based 4-way push-pull PAs combined with the Lange couplers. In this section, the design of the 4-way PA is described by utilizing the transformer-based power divider and combiner in the current mode architecture.

### 2.1. Architecture of 4-Way PA

**Figure 1:**
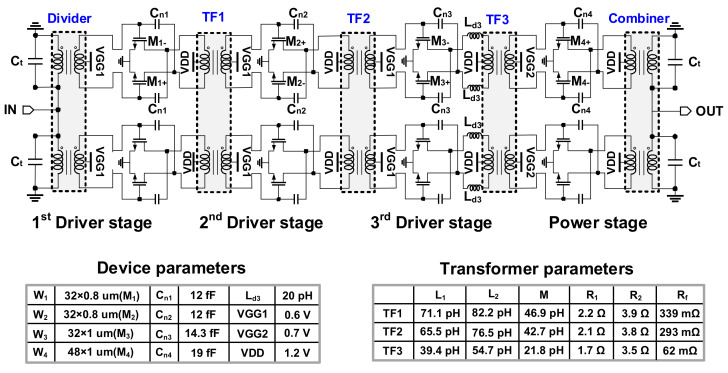
Schematic diagram of the 4-way transformer-based push-pull power amplifier used in the balanced 8-way PA.

The schematic of the proposed 4-way 4-stage PA is presented in Figure 1. It is composed of an input power divider and output power combiner in the current domain, two 2-way PAs with the push-pull pairs utilizing transformer-based interstage matching networks. For each push-pull pair for the 2-way PA, the capacitive neutralization technique was employed to enhance the impedance matching and stability of the circuit. In this architecture, two push-pull PAs are combined in the current domain with a transformer-based combiner, which can increase the effective impedance seen from each push-pull PA by twice, which eventually helps in choosing a proper output device size of the push-pull PA to improve the power efficiency and the current handling capability of the output matching network.

### 2.2. Differential PA Unit Cell

We utilized the push-pull structure with a differential pair using the capacitive neutralization technique to improve the stability factor as presented in Figure 1. This architecture eventually results in better impedance matching and increases the isolation between input and output as well as the gain of each stage. The value of the neutralization capacitor was selected to be close to the *C_gd_* of the transistor.

**Figure 2:**
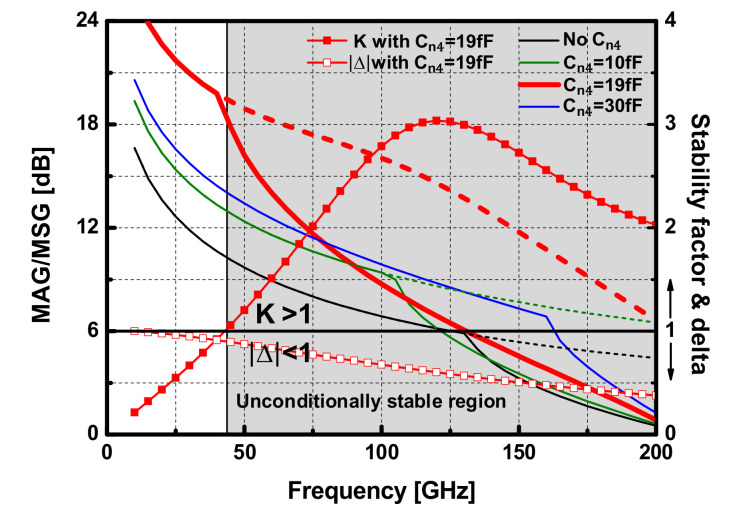
Simulated MSG (dashed lines)/MAG (solid lines) of the power stage differential pair depending on the neutralization capacitor C_n4_ and K and |Δ| with C_n4_ = 19 fF.

Figure 2 shows the MSG/MAG of the differential pair in the power stage depending on the neutralization capacitance C_n4_ over frequency. In this design, C_n4_ = 19 fF was chosen, and it is verified that the active device with the capacitive neutralization technique was stable from the extracted K and |Δ| at W-band. The gate width (W) of the first and second driver stage was *W*_1,2_ = 32 × 0.8 μm. For the transistors at the 3rd driver stage and power stage, *W*_3_ = 32 × 1 μm, and *W*_4_ = 48 × 1 μm were used, respectively. To achieve optimal capacitive neutralization for a given device size in each stage, the neutralization capacitors were set by C_n1,2_ = 12 fF, C_n3_ = 14.3 fF, and C_n4_ = 19 fF.

### 2.3. Transformer-Based Matching Network

**Figure 3:**
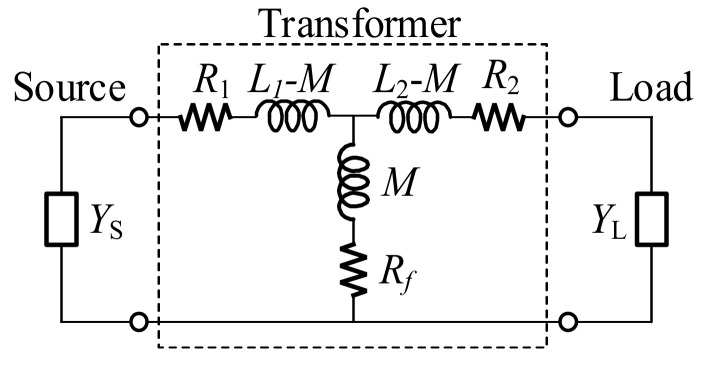
Six-parameter transformer model with general load and source.

To model an on-chip transformer (TF) for a PA design in CMOS technology, a low-frequency model with six parameters (*L*_1_, *R*_1_, *L*_2_, *R*_2_, *M*, and *R_f_*) has been used to characterize the two winding inductors and the inductive coupling, the capacitive coupling, respectively, as illustrated in Figure 3 [14,15,16]. The source and the load of the transformer can be either the 50-Ω terminal, or the gate or the drain of transistors in interstage matching network design. The coupling coefficient and the quality factors are defined as below:

(1) $$k=\frac{M}{\sqrt{L_{1}L_{2}}};\;\;Q_{1}=\frac{\omega L_{1}}{R_{1}+R_{f}};\;\;Q_{2}=\frac{\omega L_{2}}{R_{2}+R_{f}}$$

The optimum source (*Z_s_* = *R_s_ + jX_s_*) and load (*Z_L_ = R_L_ + jX_L_*) for a given transformer are given in [15], and they are written in terms of admittances as:

(2a) $$B_{s}=\frac{1}{\omega L_{1}}\cdot \frac{Q_{1}^{2}}{1+Q_{1}^{2}+k^{2}Q_{1}Q_{2}};G_{s}=\frac{1}{R_{1}}\cdot \frac{\sqrt{1+k^{2}Q_{1}Q_{2}}}{1+Q_{1}^{2}+k^{2}Q_{1}Q_{2}}$$

(2b) $$B_{L}=\frac{1}{\omega L_{2}}\cdot \frac{Q_{2}^{2}}{1+Q_{2}^{2}+k^{2}Q_{1}Q_{2}};G_{L}=\frac{1}{R_{2}}\cdot \frac{\sqrt{1+k^{2}Q_{1}Q_{2}}}{1+Q_{2}^{2}+k^{2}Q_{1}Q_{2}}$$

where *Y_S_* = *jB_S_* + *G_S_* is the source admittance and *Y_L_* = *jB_L_* + *G_L_* is the load admittance of the transformer.

The conductance and susceptance of the source and load can be transferred into the forms of parallel resistance (*R_p_*) and reactance (*X_p_*) as below:

(3) $$R_{sp}=\frac{1}{G_{s}}\cdot X_{sp}=\frac{1}{B_{s}}\cdot R_{Lp}=\frac{1}{G_{L}}\cdot X_{Lp}=\frac{1}{B_{L}}$$

For an on-chip transformer in the CMOS process, the five-parameter model with *R_f_* = 0, can properly model the electrical behavior of a winding transformer up to around 70% of its SRF with 10% of precise tolerance [16]. It is noteworthy that the intrinsic insertion loss of a TF is reduced monotonically for k^2^Q_1_Q_2_.

**Figure 4:**
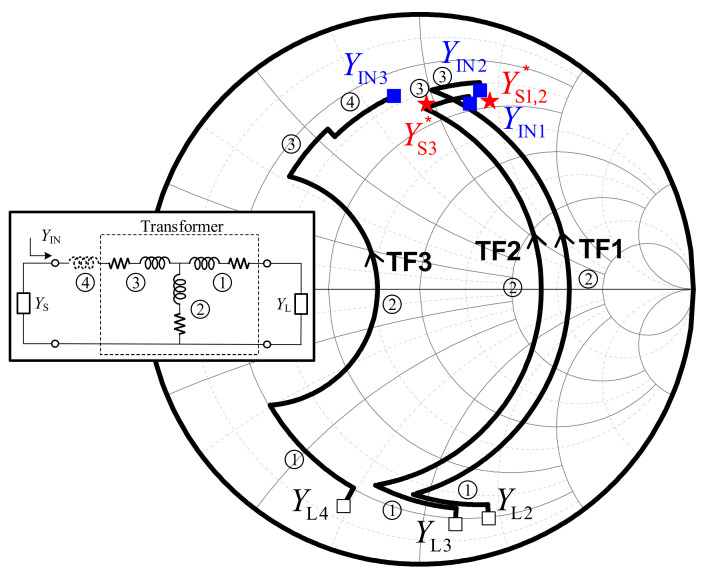
Admittance derived from 3D EM results (blue squares), the admittance from the modeled lumped components (black solid lines), and conjugate source admittance (red stars) of the TF1, TF2, and TF3 at each interstage in the 4-way PA.

In designing the interstage matching networks, transformers with a center tap were employed and named TF1, TF2, and TF3, as illustrated in Figure 1. Considering the device size and neutralization capacitors of each unit cell amplifier, interstage matching networks are designed using the lumped components model of the transformer, presented in Figure 3. The turns ratio of all the interstage transformers is 1:1, and the transformers operate well below the self-resonance frequency (SRF) to guarantee the validity of the lumped component model. In the case of TF3 at the input of the final stage, additional series inductors were applied to resonate out the drain-source capacitance. Figure 4 presents the trace at each interstage by using the lumped components model. Since the size of M1 equals M2, the conjugate source admittance is plotted at the same point. The impedance of the lumped model corresponds well with 3D EM simulated results, as presented in Figure 4.

### 2.4. Transformer-Based Parallel Combiner

**Figure 5:**
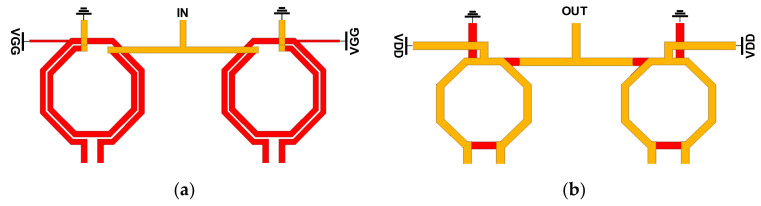
Configuration of (**a**) the divider and (**b**) the combiner at the input and output of the 4-way PA.

**Figure 6:**
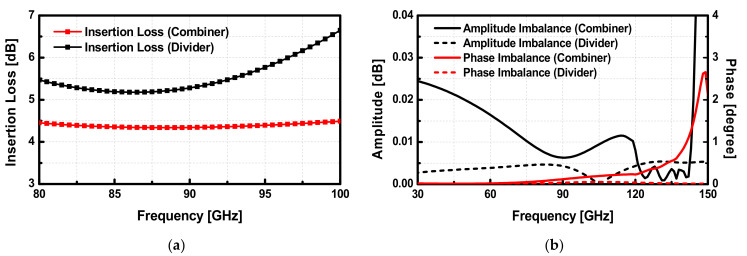
Simulated (**a**) insertion loss and (**b**) amplitude and phase imbalance of the divider and the combiner in the 4-way PAs.

In implementing the transformer-based combiner, the ultra-thick metal (UTM) with the thickness T = 3.4 μm was used for the primary coil to minimize the voltage drop in the path of VDD, while M8 was used for the secondary coil. In this structure, the input impedance of the matching network at each differential port of the primary coils is well balanced, even though the secondary coil is a single-ended structure connected to the ground. The 3D EM model of the input divider and output combiner is described in Figure 5a,b, respectively. All the simulations of the passive components were performed with Ansoft HFSS. The diameter of the divider and the outside coils of the combiner are 52 μm and 46 μm, respectively. For the transformer-based divider in Figure 5a, the primary coils have a larger self-inductance compared to the secondary coil as the diameter of the secondary coil is 39 μm. To improve the coupling factor k for the transformer, broadside-coupled coils were implemented on the M8 layer, which has a smaller minimum spacing rule than the UTM layer. The simulated insertion losses of the combiner and the divider are shown in Figure 6a. In implementing the output parallel combiner for each 4-way push-pull PA, vertical-coupled transformers were used to combine output signals at the coils. This on-chip vertical-coupled structure is advantageous to combine signals with low insertion loss, as it can achieve a very small distance between the primary and secondary coils. Owing to the symmetry in the layout, the parallel combiner with the current mode is highly balanced. To verify the electrical symmetry of the transformer-based combiner and divider, the amplitude and the phase imbalance of each component are simulated, which shows almost 0 dB and below 1-degree imbalance below 140 GHz, as presented in Figure 6b. The small amplitude and phase imbalances are caused by the paths for cross-coupled capacitors in different layers.

**Figure 7:**
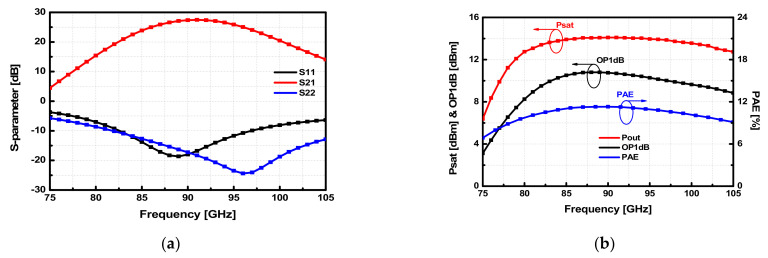
Simulation results of (**a**) S-parameters and (**b**) large signals of the W-band 4-way PAs.

The output power matching has been performed using the Load-pull analysis with Harmonic Balanced (HB) simulation. The differential optimum impedance *Z_opt1diff_* and *Z_opt2diff_* of the power stage are extracted using load-pull simulations, and each input impedance at differential ports of the output matching network is matched to *Z_opt1diff_* and *Z_opt2diff_*. The simulated S-parameters of the designed 4-way PA are shown in Figure 7a. The 4-way PA achieves a peak gain of 27.5 dB at 91 GHz with a 3-dB gain bandwidth of 11 GHz (85.5–96.5 GHz). The input and output return losses are larger than 10 dB within the 3-dB gain bandwidth. Also, the large-signal results of the 4-way PA were simulated. The simulated output power and the 1-dB compressed output power () are 14.2 dBm and 10.8 dBm at 90 GHz, respectively. The maximum power efficiency of the 4-way PA is simulated to be 11% at 90 GHz. In the simulations of 4-way PA, the input and output port impedance were set to 35 Ω, which is a characteristic impedance of the transmission line in the Lange coupler. The simulation and layout of the designed PA were performed with Cadence Virtuoso. Figure 7b shows simulated results of the large-signal performance versus frequency.

## 3. Design of Balanced 8-Way Power Amplifier

### 3.1. W-Band Microstrip Lange Coupler

The advantage of utilizing a balanced power amplifier configuration is its wideband matching at input and output. Moreover, it guarantees a wide range of stability since the reflected powers from the impedance mismatches are dissipated in the 35 Ω termination of the isolation port at the coupler. Through the balanced structure, the flatness can also be improved as the unbalanced output power from the mismatches of the two 4-way PAs must be dissipated in the termination as well [17]. Owing to this characteristic, a balanced PA is ideal for a high-precision FMCW radar sensor.

To achieve the balanced architecture with an improved *P_sat_* for a wideband operation, the Lange couplers were employed to combine the output powers from each 4-way PA. It is noteworthy that the size of the Lange coupler becomes comparable to that of the couplers with lumped components at W-band. Moreover, the insertion loss of the transmission-line-based couplers becomes even smaller than its counterparts at 90 GHz. The designed Lange coupler utilizes the UTM layer as a coupled line to mitigate the insertion loss (IL).

**Figure 8:**
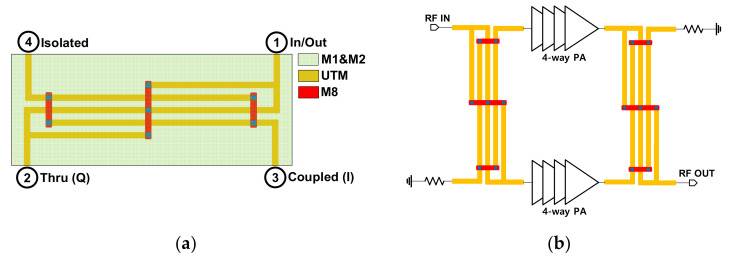
(**a**) Structure of the microstrip Lange coupler and (**b**) block diagram of the balanced PA with the Lange coupler.

To construct the Microstrip-line, M1 and M2 layers were stacked together with via to form a solid ground plane, and the UTM (ultra-thick metal layer) layer was used as a signal line. For the Lange coupler, the bridges were formed with the M8 layer as illustrated in Figure 8a. The length of the hybrid coupled combiner is *λ_g_*/4 ≈ 450 μm for 90 GHz. It should be noted that the reference impedance was chosen to be 35 Ω considering the limited minimum width of the UTM layer in implementing the coupled line of the Lange coupler.

**Figure 9:**
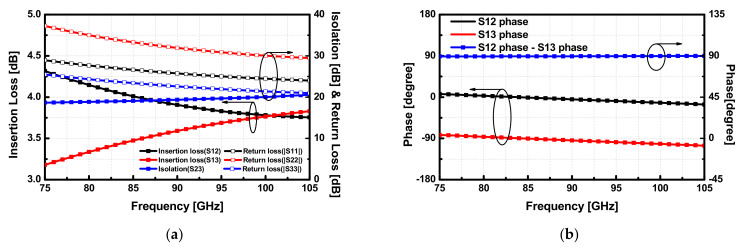
Simulation result of the (**a**) insertion loss, isolation and (**b**) phase response for the designed Lange coupler.

Even though the input and output VSWR of the whole PA highly rely on that of the Lange coupler, the return loss (RL) from the mismatch between 35 and 50 Ω load is still good enough (RL≈19 dB). Hence, the load impedance of the 4-way PA was also designed to be 35 Ω. Moreover, a reference impedance of 35 Ω in the Microstrip-line features wide signal paths that allow more current to flow [7]. As illustrated in Figure 8b, the implemented 8-way PA forms a balanced structure with two 4-way four-stage push-pull PAs utilizing the input and output Lange couplers with its high coupling feature, owing to the multiple fingers. The 4-way PAs were implemented with two transformer-based push-pull PAs using transformer-based combiners and dividers, as presented in Figure 1. The simulated magnitude and phase of the S-parameters for the designed Lange coupler are shown in Figure 9a,b. Transistors in two internal paths were placed away from DC pads, and it can increase common-mode inductance due to relatively lengthy supply lines. Herein, bypass capacitors were placed not only around DC pads, but also between two quadrature paths in the coupler.

### 3.2. Robustness to Load Mismatch

**Figure 10:**
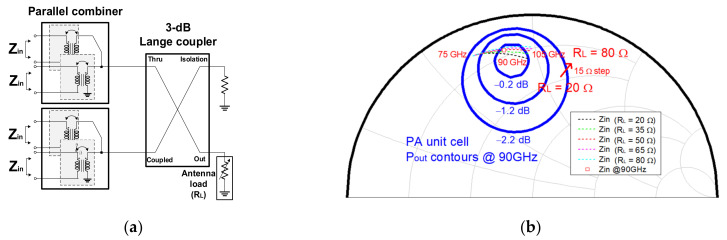
(**a**) Output matching network and (**b**) Output power contours and the input impedance of the output matching network with the load variations from 20 to 80 Ω at 90 GHz.

In this design, the extracted differential optimum load impedance *Z_optdiff_* = 11.3 + j43 Ω at 90 GHz is matched from a 50 Ω load. However, the load impedance cannot exactly be 50 Ω, in practice, since the connection between an antenna at the output and the PA causes load variation. To check the robustness to the load variations, the impedance of the load *R_L_* was varied from 20 to 80 Ω (±60% variations) in the simulation. Then, we observed the output power, output return loss, and the input impedance variation of the transformer-based combiner preceding the Lange coupler. The output matching network and unintentionally variable antenna load are shown in Figure 10a. The load impedance does not affect much the input impedance of the output matching network, owing to the balanced structure of the designed PA as shown in Figure 10b.

**Figure 11:**
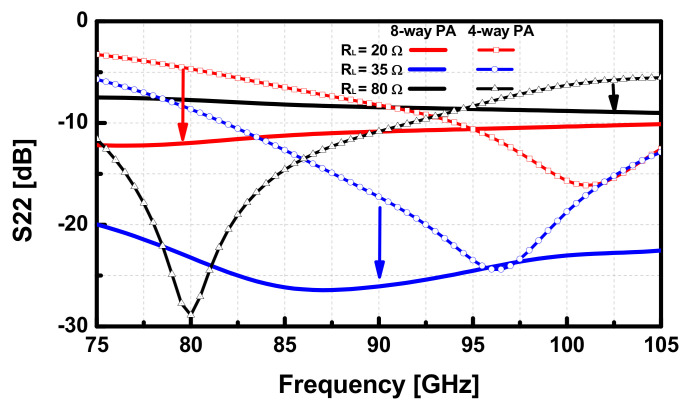
Simulated output return loss (S22) of the 8-way PA and 4-way PA with the load variations.

The arrows in Figure 11 indicate the improved output return loss (RL) of the 8-way PA compared with that of the 4-way PA. Meanwhile, the output RL of the 8-way PA with an 80 Ω load achieved the output RL better than the acceptable level (>8 dB) in the working range between 75 and 105 GHz; the S22 of the 4-way PA was distinctively shifted depending on the load variations, which resulted in 5 dB of RL in the operating band. In the load variations, the PA achieved the minimum *P_sat_* of 15.7 dBm with the 80 Ω load and the maximum *P_sat_* of 16.2 dBm with the 50 Ω load at 90 GHz. As such, the simulated output power results show that the Lange coupler compensates for the load variations, which effectively mitigates the performance degradations of the PA from the load variations.

## 4. Measurement Results

**Figure 12:**
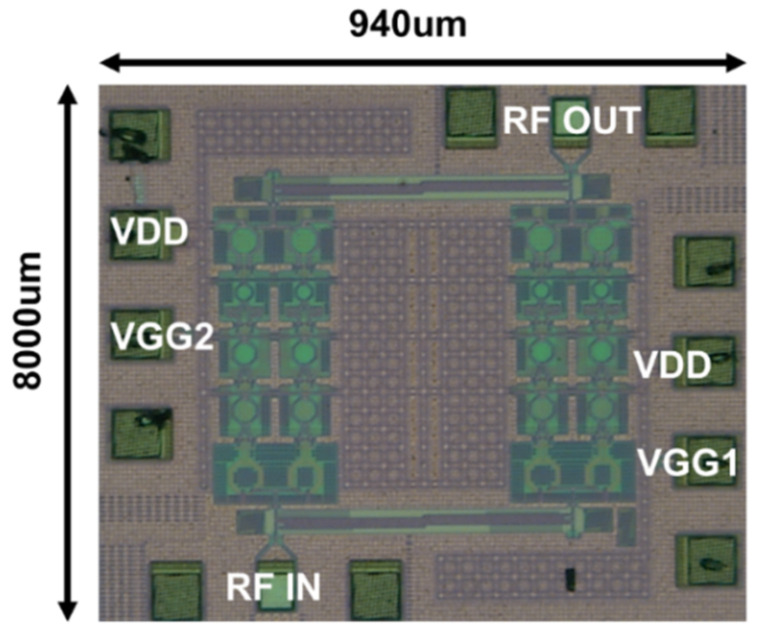
Chip photograph with the size of 0.94 mm × 0.8 mm including RF pads and DC pads.

The chip size of the implemented PA is 0.94 × 0.8 mm^2^, including RF pads and DC pads. Without RF pads and DC pads, the size of the core area is 0.62 × 0.62 mm^2^. The chip photograph of PA is presented in Figure 12. For the measurement of S-parameters of the implemented balanced PA, a vector network analyzer N5224A (Keysight, CA, USA) combined with an extension module (75–110 GHz) was used with an on-wafer probe station, and on-wafer setup was calibrated with a CS-5 calibration kit.

**Figure 13:**
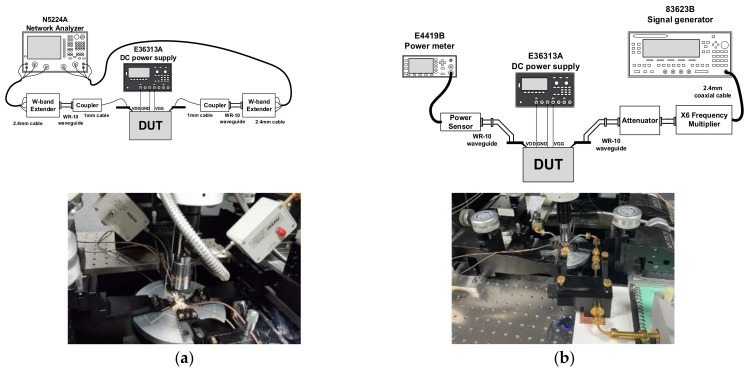
Measurement setup of the proposed W-band PA (**a**) S-parameters and (**b**) the large-signal performance.

The implemented PA consumes a DC-current of 370 mA from a 1.2-V supply. In measuring the large-signal performance, the W-band input signal was generated using a ×6 frequency multiplier and an X-band signal generator 83623B (Agilent, CA, USA), A W-band attenuator QAD-W00000 (Quinstar, CA, USA) was placed at the output of the frequency multiplier to control the input power. The output power of the PA was measured using a power meter E4419B (Agilent, CA, USA) and a W-band power sensor W8486A (Agilent, CA, USA). The setup for the S-parameters and the large-signal measurement at W-band are illustrated in Figure 13a,b, respectively.

**Figure 14:**
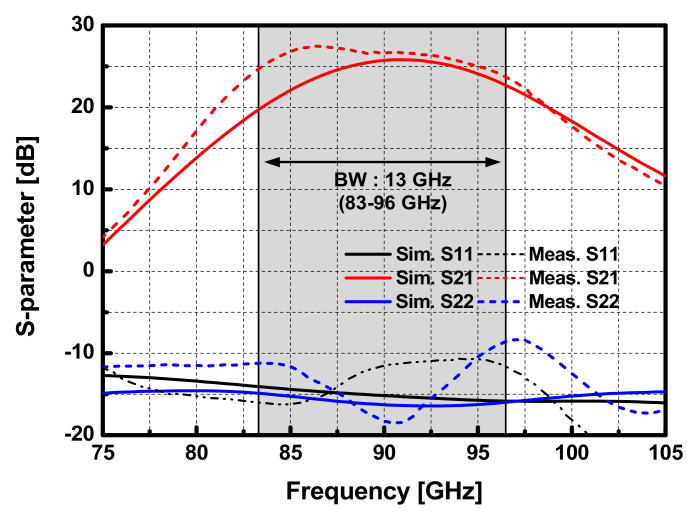
Measurement and simulation results of S-parameter of the W-band 8-way PA with a 50 Ω load.

**Figure 15:**
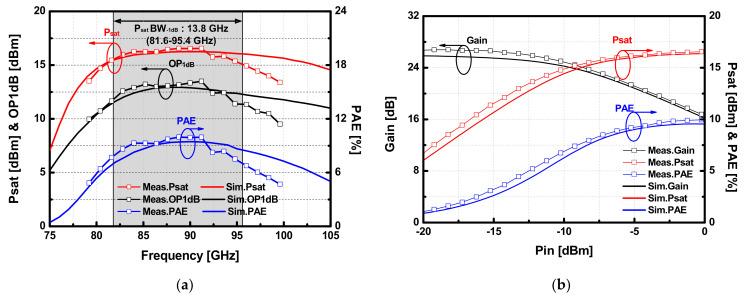
Results of the large-signal measurement of the W-band 8-way PA versus (**a**) frequency (**b**) input power at the center frequency (90 GHz).

The measured S-parameters of the balanced PA are presented in Figure 14. The peak gain improvement of 27.4 dB was observed at 86.4 GHz, and the 3 dB gain bandwidth of the balanced PA was measured to be 13 GHz (83–96 GHz). Owing to the broadband feature of the Lange coupler, the input and output return loss are above 10 dB from 75 GHz to 105 GHz, except for around 97 GHz. Figure 15a,b show results of the large-signal measurement over the frequency and input power, respectively. The implemented PA achieved the peak *P_sat_* of 16.5 dBm, OP_1dB_ of 13.3 dBm, and PAE was 9.9% at 90 GHz. Within the 3-dB gain bandwidth, the designed balanced 8-way PA performs *P_sat_* higher than 14.9 dBm, where the maximum variation of 1.6 dB is observed within the 3-dB gain bandwidth. To demonstrate the output power flatness, which is advantageous for the application of PA in high-precision FMCW radars, the 1-dB power bandwidth *(P_sat_BW*_-1dB_) and 3-dB power bandwidth (*P_sat_BW*_-3dB_) were evaluated from the measurement results, as shown in Figure 15a. *P_sat_BW*_-1dB_ is 13.8 GHz (81.6–95.4 GHz), and *P_sat_W*_-1dB_ is 20.4 GHz (79.2–99.6 GHz). Even though the power bandwidths were degraded from estimated values in simulation due to the low *P_sat_* above 94 GHz in the measurement, the implemented balanced PA achieved a broad power bandwidth representing the availability of high-precision FMCW radars.

Table 1 summarizes the performance of the implemented PA in this work and other recently reported W-band CMOS PAs. Both PAs in [5,6] utilized transmission-line (T-line)-based combiners. The PA in [5] achieved a 38 GHz of bandwidth, and the 16-way PA in [6] performed the highest output power among W-band PAs. However, it is noticed that the PAs with T-line combiner had relatively lower PAE due to the lossy output matching network. Works in [9,10,11,18] utilized compact transformer-based combiners, and they achieved the saturated output power higher than 14 dBm with small area occupancy. However, with this configuration, *P_sat_BW*_-1dB_ is relatively lower than other reported PAs. Meanwhile, our proposed balanced PA features better 1-dB and 3-dB power bandwidth by efficiently combining two transformer-based push-pull PAs with Lange couplers. The implemented 8-way PA achieved the highest FoM and FoM_BW_ among the recently reported CMOS PAs operating above 90 GHz to date.

## 5. Conclusions

We demonstrated a W-band balanced power amplifier (PA) that achieves a 20.4-GHz of 3-dB power bandwidth with +16.5 dBm of the peak saturated output power (*P_sat_*) and 9.9% of the peak power added efficiency (PAE) in 65nm CMOS technology. To achieve the broadband 8-way PA at 90 GHz, each transformer-based 4-way PA was implemented by combining two push-pull PAs in the current mode. By utilizing on-chip Lange couplers as balancing hybrids, the two transformer-based 4-way PAs were effectively combined for the wideband operations. The implemented 8-way PA demonstrated a wideband operation to be used for high-precision FMCW radars with the highest FoM among the recently published s CMOS PAs at the 90 GHz regime.

## Figures and Tables

**Table 1:** Comparison table of CMOS power amplifiers.

	This	[5]	[6]	[9]	[10]	[11]	[18]	[19]
Freq (GHz)	83–96 @90	77–110 @87	75–100 @90	85–100 @94	101–117 @109	100–117 @109	73–89 @81	75–90 @80
VDD (V)	1.2	1.2	1.2	1.8	2/1.2	1.2	2.5	2
Gain (dB)	26.7	18	12.5	13	14.1	20.3	16.1	11
$P_{\mathrm{sat}}$(dBm)	16.5	14	18	14	14.8	15.2	18	12.4
$P_{\mathrm{sat}}BW_{-1\mathrm{dB}}$(GHz)	13.8	38 † (*OP*_1dB_)	12	>11 ‡	>7 ‡	>9 ‡	N/A	>7 ‡ (OP_1dB_)
$P_{\mathrm{sat}}BW_{-3\mathrm{dB}}$(GHz)	20.4	N/A	N/A	15	>10 ‡	>16 ‡	N/A	>10 ‡(OP_1dB_)
${\mathrm{OP}}_{1\mathrm{dB}}$(dBm)	13.3	12	17.5	10.3	11.6	12.5	12.9	12
PAE (%)	9.9	4.5	9	4	9.4	10.3	12.6	14.2
Way	8	4	16	4	4	4	4	1
Size (${\mathrm{mm}}^{2})$	0.752	0.57	0.82	0.24	0.322	0.343	0.21 (core)	0.321
FoM *	92.2	77.3	79.1	72.5	79.4	86.4	83.2	73.0
${\mathrm{FoM}}_{\mathrm{BW}}$**	2420.0	204.8	237.2	28.3	127.3	677.2	419.7	37.3
Topology	4 stage CS + BA	6 stage CS	3 stage CS	3 stage CC	2 stage CC + 1 stage CS	4 stage CS	2 stage CC	2 stage CC
Process	65 nm	65 nm	65 nm	65 nm	65 nm	65 nm	55 nm	45 nm SOI

† Simulated result. ‡ Graphically estimated from measurements. * FoM = $P_{sat}$ [dBm] + Gain[dB] + 10log(PAE[%] × $f_{c}^{2}\left[\mathrm{GHz}\right]$ ). **${\;\mathrm{FoM}}_{\mathrm{BW}}$ = $P_{sat}$ [W] × Gain × PAE[%] × $f_{c}^{2}\left[\mathrm{GHz}\right]$ × BW_gain_[%] [20]. CS: Common source, CC: Cascode, BA: Balanced amplifier.

## Data Availability

Not applicable.
